# Nano protective membrane coated wheat to resist powdery mildew

**DOI:** 10.3389/fpls.2024.1369330

**Published:** 2024-03-21

**Authors:** Huilan Zhang, Meng Yuan, Yameng Gao, Pengfei Su, Huiling Jia, Caiguo Tang, He Meng, Lifang Wu

**Affiliations:** ^1^ The Center for Ion Beam Bioengineering & Green Agriculture, Hefei Institutes of Physical Science, Chinese Academy of Sciences, Hefei, Anhui, China; ^2^ Zhongke Taihe Experimental Station, Taihe, Anhui, China; ^3^ CNSIG Anhui Hongsifang Fertilizer Co., Ltd., Hefei, Anhui, China; ^4^ Institute of Hefei Artificial Intelligence Breeding Accelerator, Hefei, Anhui, China; ^5^ School of Life Sciences, University of Science and Technology of China, Hefei, Anhui, China; ^6^ School of Life Sciences, Anhui Agricultural University, Hefei, China

**Keywords:** wheat, powdery mildew, KTP (kaolin - TiO2-NPs - liquid paraffin), disease index, green prevention and control

## Abstract

The plant pathogenic fungus *Blumeria graminis* f. sp. *tritici* infects wheat and reduces its yield. The policy of reducing fertilizer and biocide use in sustainable agriculture has prompted researchers to develop more green and efficient management strategies. In this study, a novel nanoprotective membrane (kaolin-nano titanium dioxide-liquid paraffin, referred to as KTP) that could effectively prevent powdery mildew of wheat was prepared by using 1 g/L kaolin, 2 g/L nanotitanium dioxide and 8% (v/v) liquid paraffin. The prevention and control effects of KTP spraying in advance in the pot and field experiments were 98.45% and 83.04%, respectively. More importantly, the weight of 1000 grains of wheat pretreated with KTP was 2.56 g higher than that of wheat infected with powdery mildew, significantly improving wheat yield. KTP delayed the germination of powdery mildew spores on the leaf surface, and inhibited the formation of mycelia. In addition, KTP did not affect the growth of wheat or the survival of earthworms. KTP nanoprotective membrane are a green and safe prevention and control materials that are which is expected to be widely used in agriculture to control wheat powdery mildew.

## Graphical Abstract

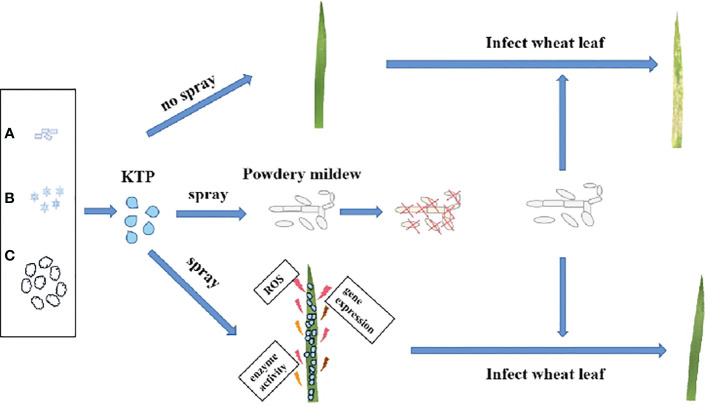

## Highligts

- The powdery mildew inhibitor KTP is composed of 1 g/L kaolin, 2 g/L nanotitanium dioxide and 8% (v/v) liquid paraffin.
- KTP is formed by hydrogen bonding and electrostatic attraction between components.
- The prevention effects of KTP in the pot and field experiments were 98.45% and 83.04%, respectively.
- The yield of wheat increased significantly after the control of powdery mildew by KTP.
- KTP delayed the germination of powdery mildew spores on the leaf surface and inhibited the formation of mycelia.

## 1 Introduction

Wheat is one of the most important food crops worldwide, and its sustainable production directly affects the stable development of a country (Yinghui et al., 2016). Wheat powdery mildew is a wheat leaf disease caused by the obligate parasitic ascomycetous fungus *Blumeria graminis* f. sp. *tritici* (*Bgt*) that is endemic in warm and humid regions of the world, such as China, the United Kingdom, Germany, Japan, and the southeastern United States (Saari and Wilcoxson, 1974; Merchan and Kranz, 1986; Mandal et al., 2014), but especially in northern Europe (Reignault et al., 2001). Under nutrient-rich conditions, *Bgt* spores germinate and produce appressoria, form penetration pegs, enter host cells to form haustoria, absorb nutrients from host cells for the rapid growth of secondary hyphae, and, finally, form colonies (Zheng et al., 2020). Colonies cover the surface of wheat leaves, resulting in chlorophyll degradation and a decrease in the photosynthetic rate (Kuckenberg et al., 2009; Walters et al., 2008; Wright et al., 1995). Therefore, powdery mildew destroys the vegetative growth of the aboveground parts of wheat plants directly, causing an annual yield loss of 14 to 30% (Griffey et al., 1993), and is one of the most destructive diseases in the global wheat industry (Reignault et al., 2001).

To date, the control of powdery mildew relies mainly on the cultivation of disease-resistant plant varieties and the spraying of pesticides. Planting disease-resistant varieties is considered to be one of the most cost-effective ways to prevent and control of wheat powdery mildew (Summers and Brown, 2013; Xin et al., 2012). However, pathogenic bacterial variants alter the physiological effects of antimicrobial agents on plants, causing most cultivated plant varieties to lose resistance after 3-5 years (Vallavieille-Pope et al., 2012). The use of fungicides for many years has led to many problems, such as pathogen resistance, induced plant toxicity and environmental pollution. Therefore, manufacturers urgently need to develop new green and safe preparations for the prevention and control of wheat powdery mildew in the field.

Currently, the practical application of nanotechnology in the agricultural industry has received widespread attention (Santhoshkumar et al., 2014). And NPs have broad potential for agricultural application (Ali et al., 2014). However, research on the management of wheat powdery mildew with nanomaterials is relatively limited, and the expected effect has not been achieved. Moreover, the lack of a control mechanism limits the application of nanomaterials in wheat powdery mildew management. Here, we explored the feasibility of using nanotitanium dioxide (TiO_2_-NPs) and kaolin as nanopesticides to control wheat powdery mildew. Wherein, TiO_2_-NPs has been widely used for its antibacterial and antifungal properties (Santhoshkumar et al., 2014; Waghmode et al., 2019; Rodríguez-González et al., 2019), which can help plants resist some bacterial and fungal diseases (De Filpo et al., 2013; Javed et al., 2020; Owolade and Ogunleti, 2008). Kaolin is a nonmetallic mineral with good plasticity that can be used in pesticide production (Cantore et al., 2009). Then, the novel nanocomposite was obtained by combining it with the modifier liquid paraffin.

In this study, 1 g/L kaolin, 2 g/L nanotitanium dioxide, and 8% (v/v) liquid paraffin were used to prepare the nanoprotective membrane (kaolin- nanotitanium dioxide -liquid paraffin, referred to as KTP). We explored the mechanism by which KTP controls wheat powdery mildew at the molecular, physiological and biochemical levels. KTP plays an important role in regulating the expression of the pathogenesis-related protein-encoding gene *PR1*, the oxalate oxidase gene (*OXO*) and the chitinase gene (*CHI1*). We found that KTP delayed the germination of powdery mildew spores on the leaf surface and inhibited mycelium formation. The physicochemical properties of KTP showed that it was formed by hydrogen bonding and electrostatic attraction among the components, and KTP could be uniformly applied to the surface of wheat leaves with good hydrophobicity and thermal stability, meeting the needs of the field. Furthermore, spraying KTP in the field could effectively prevent wheat powdery mildew incidence and reduce the loss of wheat yield and quality. The above results showed that the nanoprotective membrane could effectively control wheat powdery mildew. And KTP has the ability to control wheat powdery mildew on a large scale. We believe that this new plant protection material will provide farmers with a novel management strategy to prevent powdery mildew, which will have a positive impact on sustainable agricultural production.

## 2 Materials and methods

### 2.1 Materials

Kaolin (CAS:1332-58-7, 1250 mesh) was purchased from Yuejiang New Materials Co., Ltd (Guangzhou, Guangdong, China). Nanotitanium dioxide (TiO_2_-NPs, CAS:13463-67-7, 99.8% anatase type) was purchased from Shanghai Aladdin Biochemical Technology Co., Ltd. (Shanghai, China). Liquid paraffin (CAS: 8042-47-5) was obtained Sangon Biotech (Shanghai) Co., Ltd (Shanghai, China). The other chemicals used in this work were all of analytical reagent grade and purchased from Sinopharm Chemical Reagent Co., Ltd.

The winter wheat (*Triticum aestivum L*.) variety Bainong 207 (provided by Professor Hao Chenyang of the Institute of Crop Sciences, Chinese Academy of Agricultural Sciences), which is sensitive to powdery mildew infection, was used for all the experiments in this study. The grains of Bainong 207 that were of uniform in size and not damaged were selected and planted in pots containing rich nutritive soil (diameter = 10 cm), with 20 seeds in each pot. The wheat plants were grown in a constant temperature and light incubator with a light intensity of 300 μmol photons m^-2^s^-1^, 22°C/20°C, 16 h light/8 h dark cycle, and 85% relative humidity.

### 2.2 Preparation of KTP

A total of 0.1 g of kaolin, 0.2 g of TiO2-NPs and 8 mL of liquid paraffin were added, after which the volume was brought to 100 mL with deionized water. The resulting suspension was stirred at 1500 rpm/min for 2 h, followed by ultrasonic treatment for 1 h. The resulting mixture was named KTP (kaolin - TiO_2_-NPs - liquid paraffin) and was incubated at room temperature condition.

### 2.3 Characterization of KTP

The prepared sample was directly analyzed via contact angle (CA), Fourier transform infrared (FTIR) spectroscopy, the thermal gravimetric analysis (TGA), differential thermal analysis (DTA), zeta potential analysis and scanning electron microscopy -energy dispersive spectrometer (SEM-EDS).

The contact angle (CA) (JY-82, Dataphysics Co., Germany.) was detected according to the sessile drop method at room temperature. Detailed steps are as follows: appropriate amounts of kaolin and titanium dioxide were tabletted, and then a drop of water was dropped directly above the surface of the slide containing each sample tablet and photographed during the process, and the contact angle between the water droplet and the surface of each sample was measured by goniophotometric method. Subsequently, the contact angle of KTP was determined by applying it uniformly to the slide surface.

The samples were also characterized by Fourier transform infrared spectroscopy (FTIR) (iS10, Nicolet Co., U.S.A.) in a KBr pellet at ambient temperature, wherein the sample content in KBr was 0.5%. For each sample, 32 scans were recorded in a 400–4000 cm^−1^ spectral range at a resolution of 4 cm^−1^. The infrared spectra of kaolin and titanium dioxide nanoparticles were collected. Then, the ATR (Attenuated Total Refraction, ATR) accessory was placed in the optical path of the spectrometer, and 1 drop of liquid paraffin and KTP were pipetted onto the crystal surface of the ATR accessory, and the infrared spectra of the liquid paraffin and KTP were collected with an air background as a blank control.

The thermal gravimetric analysis and differential thermal analysis were performed by a thermogravimetric analyzer (TG-DSC) (STA 449F3, NETZSCH Co., Germany.) at a scan rate of 10 °C/min from room temperature to 800°C in nitrogen. And used the scanning electron microscope (SEM) (Sirion 200, FEI Co., U.S.A.) to observe the morphology of the leaves.

Scanning electron microscope and energy dispersive spectrometer(SEM-EDS)analysis of KTP nano protective membrane distribution on leaf surface. Detailed steps are as follows: wheat leaves of normal growth for 7 days, and the following 4 different treatments: normally grown wheat leaves (CK group), wheat leaves sprayed with KTP (KTP group), wheat leaves inoculated with Bgt after KTP spraying (KTP + Bgt group), and wheat leaves inoculated with Bgt (Bgt group) were carried out. After 144 h, suitable size of leaves were cut and stuck directly to the conductive adhesive and sprayed gold for 30 s. Subsequently, a scanning electron microscope (SEM) was used, in which an accelerating voltage of 15 kV was selected for the energy spectrum, and a detector was used to analyze the surface morphology of the samples as well as the elemental distribution.

The charged nature of the components was analyzed using a zeta potential analyzer (ZS90, Malvern Co., England.). The samples of kaolin, titanium dioxide, liquid paraffin and KTP were placed in deionized water and ultrasonicated for 5 min, and the average value of each group of samples was taken from three measurements.

### 2.4 Inoculation and control of powdery mildew

Bainong 207 wheat seeds were planted into pots (DI = 10 cm) and placed in a constant -temperature and constant-light incubator. After 7 days of culture in an incubator, sufficient amounts of powdery mildew fungus were cultured for inoculation. Two different treatment methods were employed: one was sprayed with KTP composites which wre subsequently inoculated with powdery mildew after drying. The other was sprayed with the KTP complex 48 hours after infection. The incidence of wheat powdery mildew and seedling growth index (plant height, fresh weight and dry weight) were investigated after 7 days of continuous culture in a light incubator. Moreover, the normal-growing wheat without treatment and that sprayed with KTP were analyzed. The wheat powdery mildew disease index DI was calculated as follows: $DI=\sum_{t=1}^{n}\left(xi*si\right)/\sum_{t=1}^{n}xi*I*100$ , where *i* is the incidence level (0%-100%), *xi* is the number of leaves with incidence level i, *si* is the severity value of incidence level *i*, and *I* is the incidence rate.7

### 2.5 Powdery mildew spore germination growth experiment

The wheat plants were subjected to the following 4 different treatments: normally grown wheat leaves (CK group), wheat leaves sprayed with KTP (KTP group), wheat leaves inoculated with *Bgt* after KTP spraying (KTP + *Bgt* group), and wheat leaves inoculated with *Bgt* (*Bgt* group). All the samples were collected in 3 biological replicates at 7 different time points (12,24.48,72,96,120,144 h) after powdery mildew inoculation. After sampling, the wheat leaves in each group were cut into 3 cm long segments, which were subsequently immersed in an ethanol/acetic acid solution (1:1, v/v) and placed at room temperature for 24 h until decolorization. After complete decolorization, the translucent leaves were washed in an aqueous solution of lactic acid and glycerin (lactic acid/glycerin/water, 1/1/1, V/V/V) for 48 h. After complete cleaning, the leaves were dyed by immersing them in 0.6% (W/V) Coomassie Bright Blue R-250 methanol solution for 1 min. Subsequently, the leaves were gently washed with deionized water to remove the excess dye. Finally, the treated leaves were preserved in an aqueous solution of lactic acid and glycerin for microscopic observation.

In addition, the number of powdery mildew spores that formed mature mycelia was counted at 48 h, and 200 spores were counted each time.

### 2.6 Changes in gene expression in leaves

Wheat leaves from the 4 different treatment groups were collected at each time point, with three biological replicates each time. After sampling, each group of wheat leaves was cut into 2 small sections, each weighing 0.5 g, placed in a centrifuge tube equipped with steel balls, transferred to liquid nitrogen for freezing, and placed in a -80°C freezer for subsequent physiological biochemical and real-time fluorescence quantitative PCR (RT–qPCR) detection.

Total RNA was isolated from the samples using a Plant RNA Isolation Kit (Omega, R6827) according to the manufacturer’s instructions. Isolated RNA samples were reverse-transcribed into cDNA using TransScript One-Step gDNA Removal and cDNA Synthesis SuperMix (TransGen Biotech, AT311) following the manufacturer’s protocol. Each cDNA sample was diluted 32X with double distilled water. RT‒qPCR was performed using a Roche Light Cycler 96 following the standard protocol; three biological replicates were used for RT‒qPCR. The housekeeping gene actin (ACT) was used as the internal control and the relative expression of the target genes was calculated using the 2^-ΔΔCT^ method. The primer sequences for real-time quantitative reverse transcription polymerase chain reaction are detailed in **Table 1**.

**Table 1:** qRT-PCR primer sequence information.

Gene	Accession number	Forward sequence(5’-3’)	Reverse sequence (5’-3’)	Gene description
*OXO*	M21962.1	CAGGGTCGTGG AACTTCTCAAG	TTATCATTTCAG GGAAGGCTCCTA	Oxalate oxidase (Tayeh et al., 2015)
*PR1*	HQ848391	CATGCACCTTCG TATGCCTAACT	TGGCTTATTAC GGCATTCCTTT	Pathogenesis-related protein 1 (Tayeh et al., 2015)
*CHI1*	AB029934	GCCACGTCCCC ACCATACTAT	CCGGCAAGAT CGTAGTTGGA	Class 1 basic chitinase (Tayeh et al., 2015)
*Act*	AB181991	AACCTTCAGT TGCCCAGCAA	TGTTCGACCG CTGGCATAC	Actin (26)

### 2.7 Field trial

A large number of powdery mildew fungi were cultivated in an artificial climatic chamber, and the powdery mildew spores on the leaves were rinsed off with sterile water, and the powdery mildew spore suspension with a concentration of 1000 U/mL was adjusted to be used for inoculation of wheat powdery mildew fungi in the field. The experimental field located in Science Island (31°54’N, 117°10’E) with planting Bainong 207 of wheat variety. The experimental areas were divided to 3 plots. Each treatment area consisted of 5 rows (2 m per row, 0.2 m between rows), and each treatment was set up with three replicates. In the evening of wheat flowering, spray the leaf surface with KTP nanofilm, tebuconazole, and powdery mildew spore suspension (1000 U/mL) respectively. The specific methods are as follows: a. Bgt + KTP group: spray the powdery mildew spore suspension first, and 2 days later, spray KTP nanofilm; b. KTP + Bgt group: spray KTP nanofilm first, let it dry naturally, and then spray the powdery mildew spore suspension; c. Tebuconazole + Bgt group: spray the pesticide tebuconazole first, let it dry naturally, and then spray the powdery mildew spore suspension; d. Bgt group: spray with an equal amount of sterile water instead of KTP, let it dry naturally, and then spray the powdery mildew spore suspension; e. KTP + Bgt + KTP group: 15 days later, perform a second spray of KTP nanofilm on the KTP 3+ Bgt group. After 30 days, the disease index of powdery mildew was counted in each of the above plots, during which normal field management such as fertilization and weed control was carried out.

### 2.8 Biosafety experiment of KTP

Ten earthworms of consistent size and vitality were picked and placed in a 1000 ml rice box containing earthy materials and incubated at room temperature for 2 days. KTP was subsequently sprayed on the soil surface layer, and the control group was sprayed with an equal volume of deionized water and incubated at room temperature for 7 days to observe the survival status of the earthworms.

### 2.9 Statistical analysis

The experimental data are presented as the average of the measurements obtained from three independent assays and are expressed as the mean ± standard error of the mean. Significant differences between treatments were further analyzed using analysis of variance (ANOVA), and the significance between pairs was tested by Duncan’s test (SPSS 26.0; IBM, Somers, NY). *P* < 0.05 was considered to indicate statistical significance.

## 3 Results and discussion

### 3.1 Prevention and control of wheat powdery mildew by KTP

**Figure 1:**
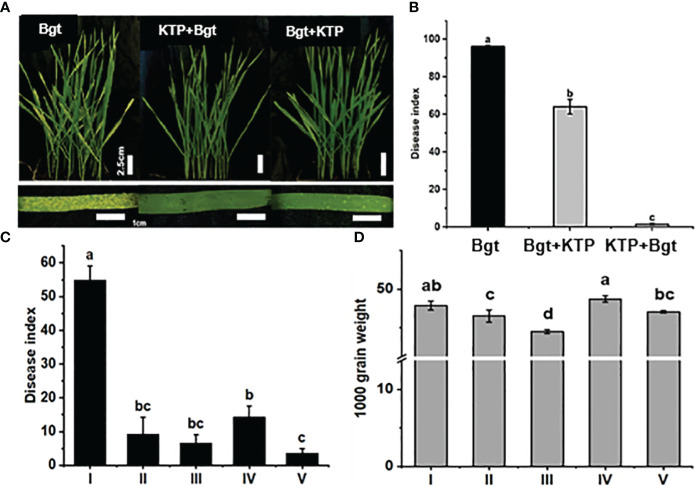
**(A)** Prevention and control of powdery mildew phenotypes in wheat cultivars under different treatments. **(B)** Disease index of wheat plants grown in pots under different treatment conditions for 7 days post infection. **(C)** Disease index of wheat in field under different treatment conditions. (I) *Bgt* group; (II) *Bgt* + KTP group; (III) KTP + *Bgt* + KTP group; (IV) KTP + *Bgt* group; (V) Tebuconazole + *Bgt* group. **(D)** Thousand grain weight of wheat under different treatments. (I) CK group; (II) Tebuconazole + *Bgt* group; (III) *Bgt* group; (IV) KTP group; (V) KTP + *Bgt* group.

Through pot experiments, we examined the efficacy of KTP in controlling powdery mildew. After 1 week, the tips of the wheat leaves in the *Bgt* group began to turn yellow and chlorotic, and powdery mildew colonies covered the leaf surface (**Figure 1A**a). Sporadically distributed white punctate plaques appeared on the surface of wheat leaves sprayed with KTP after inoculation with powdery mildew (**Figure 1A**c). Notably, no obvious colonies had grown on the surface of the leaves of wheat plants treated with KTP in advance, the leaves still appeared green, and the plants were in good growth condition (**Figure 1**Abf1). The disease index of the wheat plants from each group were also counted in this trial (**Figure 1B**). The results showed that the wheat disease index of the *Bgt* group was 96.06. Moreover, we sprayed KTP on wheat plants that had been sick for 2 days, the disease index was reduced to 32.13 and the prevention and control effect was 33.39%. The wheat disease index in the advanced KTP treatment group was 1.49, and the prevention and control effect was 98.45%, which was significantly lower than that in the *Bgt* group and sprayed KTP after inoculation with powdery mildew. This shows that the prespraying KTP prevention and control effect is the best. This result may indicate that the protective film formed on the surface of wheat leaves by spraying KTP in advance effectively hindered the germination and proliferation of powder spores on wheat leaves.

To determine the effect of spraying KTP on the control of powdery mildew in the field, we treated Bainong 207 differently and counted the disease index 30 days later (**Figure 1C**). It could be seen that the disease index of the KTP + *Bgt*, *Bgt* + KTP and Tebuconazole + *Bgt* groups were 9.33, 14.33 and 3.67 respectively. After 15 days, the KTP + *Bgt* group was sprayed with KTP for the second time, and there was no significant difference between the disease index of the KTP + *Bgt* + KTP group and that of the KTP + *Bgt* group. The disease index of the plants in each treatment proup were significantly lower than those in the *Bgt* group (55), indicating that KTP could be used to control wheat powdery mildew efficiently in the field.

Furthermore, we determined the 1000-grain weight (TGW) of wheat under different treatments (**Figure 1D**), and the results showed that there was no significant difference between the CK group and the KTP group. The TGWs of the tebuconazole + *Bgt* and KTP + *Bgt* groups were 46.53 g and 47.06 g, respectively. These values were significantly greater in the treatment groups than in the of *Bgt* group (44.5 g). It was worth noting that TGW of KTP + *Bgt* group was not significantly different from that of the CK group, which demonstrated that KTP could completely prevent wheat yield loss caused by powdery mildew infection (the TGW was reduced by 2.56 g). Therefore, KTP is suitable for large-scale promotion and application as powdery mildew prevention and control agent.

### 3.2 Characterization of the KTP complex

**Figure 2:**
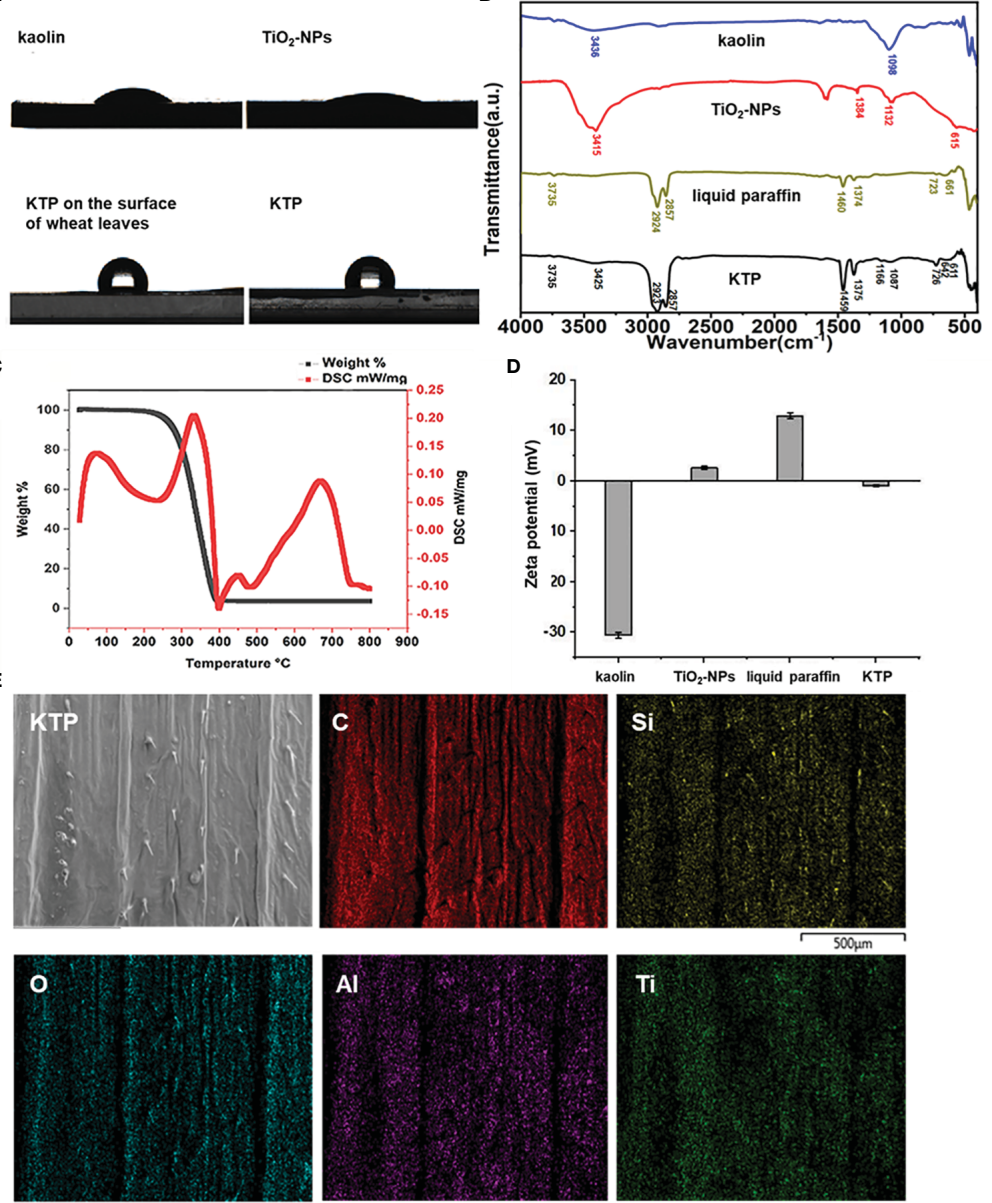
**(A)** The result of contact angle. **(B)** The results of fourier transform infrared. **(C)** The thermogravimetric curve of KTP. **(D)** Zeta potential diagram. **(E)** KTP energy spectrum scanning diagram.

The hydrophobic properties of kaolin, TiO_2_-NPs and their complex KTP were investigated. As shown in **Figure 2A**, the hydrophobic angles of kaolin and TiO_2_-NPs were 33.89° and 18.83°, respectively, while that of KTP was 102.48°. These results indicated that kaolin and TiO_2_-NPs were hydrophilic, while the nanoprotective membrane KTP was hydrophobic. The hydrophobic angle of wheat leaves treated with KTP was 106.36°, and the hydrophobic effect was stronger than that of KTP. This indicated that KTP was strongly compatible with wheat leaves, and could attach to the surface of wheat leaves better, mitigating losses caused by complex environments such as rain washing in the field.

As shown in **Figure 2B**, the peak value at 3436 cm^-1^ was ascribed to the stretching vibrations of kaolin-OH. The peak at 1098 cm^-1^ was ascribed to the stretching vibrations of kaolin Si-O-Si (Zhou et al., 2016). The peak at 3415 cm^-1^ was the -OH stretching vibration of TiO_2_-NPs, and the peak at 1384 cm^-1^ was attributed to -CH_3_. The peak at 615 cm^-1^ was ascribed to the Ti-O-Ti vibration of the TiO_2_-NPs (Irshad et al., 2020; Li-Chun et al., 2007). There was a large amount of hydrophilic -OH groups on the kaolin and TiO_2_-NP surfaces, which was also the reason why kaolin and TiO_2_-NPs exhibited hydrophilicity in the contact angle determination experiment. The peak at 3735 cm^-1^ was attributed to the free water -OH of liquid paraffin, and the peak at 2924 cm^-1^ was attributed to the symmetric and asymmetric structure of -CH_2_. The peaks at 1460 cm^-1^ and 1374 cm^-1^ attributed to -CH_3_. The peaks at 723 cm^-1^ and 661 cm^-1^ were attributed to in-plane swings of liquid paraffin -CH_2_.

The corresponding positions in the FTIR spectra of KTP could be found in the abovementioned stretching vibration of -OH, the stretching vibration of Si-O-Si, the O-Ti-O bond, the symmetry and asymmetry of - CH_2_, the in-plane swing of -CH_2_, and the in-plane swing of -CH_3_. This indicated that kaolin, TiO_2_-NPs and liquid paraffin were indeed present in KTP. Kaolin and TiO_2_-NPs were effectively modified with liquid paraffin. Compared with those in kaolin, the stretching those in of -OH and Si-O-Si in KTP were blueshifted from 3436 cm^-1^ to 3425 cm^-1^ and from 1098 cm^-1^ to 1087 cm^-1^, respectively. Compared with that of TiO_2_-NPs, the stretching vibration of KTP-OH shifted from 3415 cm^-1^ to 3425 cm^-1^ and from 1384 cm^-1^ to 1375 cm^-1^, and the O-Ti-O bond shifted from 615 cm^-1^ to 611 cm^-1^. Compared with liquid paraffin, the peak of -OH was weakened, but the peak was not changed, the -CH_3_ of KTP blueshifted from 1460 cm^-1^ to 1459 cm^-1^, redshifted from 1374 cm^-1^ to 1375 cm^-1^, the symmetric and asymmetric -CH_2_ blueshifted from 2924 cm^-1^ to 2923 cm^-1^, and the in-plane swing of -CH_2_ blueshifted from 661 cm^-1^ to 642 cm^-1^, and redshifted from 723 cm^-1^ to 726 cm^-1^. Therefore, complex KTP is formed between kaolin, TiO_2_-NPs and liquid paraffin through a large number of hydrogen bond interactions. It may ensure that KTP can form a stable protective film on the leaves, effectively resisting the invasion of powdery spores.

The TG-DSC analysis results for KTP are shown in **Figure 2C**. There was only one weight loss stage in the KTP thermogravimetric curve. The weight loss stage ranged from 273.1°C to 394°C, and the total weight loss rate was 96.3%. The reason for the quality loss was the thermal decomposition of liquid paraffin. The results of thermal analysis showed that KTP could stably persist below 273.1 °C. Therefore, KTP has good thermal stability at room temperature and could meet practical application requirements in the field.

To examine the electrical chargeability of each material, the zeta potentials of kaolin, TiO_2_-NPs, liquid paraffin, and KTP were tested in this experiment (**Figure 2D**). The results showed that, compared with that of each component, the charge of KTP was numerically lower and electroneutral, which indicated that KTP was successfully synthesized. These results indicated that kaolin, TiO_2_-NPs, and liquid paraffin further promoted the synthesis of KTP nanoprotective membranes by relying on electrostatic attraction.

To explore whether KTP was uniformly distributed on the surface of the wheat leaves, we performed energy spectral scanning analysis of C, O, Al, Si, and Ti on the surface of the wheat leaves sprayed with KTP. As shown in **Figure 2E**, the elements in KTP were distributed relatively evenly on the surface of the wheat leaves. This indicated that the KTP nanoprotective membrane could be used to uniformly cover the surface of wheat leaves, which guaranteed its disease prevention and control efficacy.

### 3.3 KTP inhibits powder spore germination

**Figure 3:**
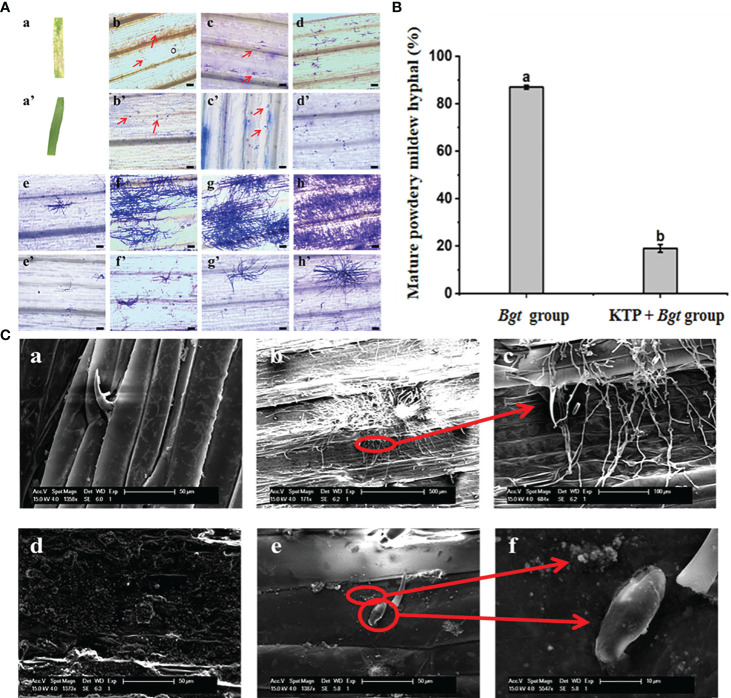
**(A)** Effect of KTP on germination and development of powdery mildew spores in wheat leaves, bar = 100 μm. (a) Occurrence of wheat powdery mildew in *Bgt* group. (a’) Occurrence of wheat powdery mildew in KTP + *Bgt* group. (b - h) and (b ‘- h’) *Bgt* group and KTP + *Bgt* group showed the spore germination and growth of powdery mildew at 12, 24, 48, 72, 96, 120 and 144 hours. **(B)** The ratio of mature powdery mildew hyphal at 48h **(C)** SEM image of KTP inhibiting conidial germination of powdery mildew. (a) Wax layer on the surface of wheat leaves; (b, c) *Bgt* group wheat leaf surface; (d) Wheat leaves sprayed with KTP; (e, f) The surface of wheat leaves pretreated by KTP.

To further investigate how KTP prevents the occurrence of powdery mildew, we conducted a spore germination growth test. After inoculation with powdery mildew for 7 days, the surface of the wheat leaves not subjected to KTP treatment was covered with a large number of powdery mildew colonies, while no powdery mildew colonies formed on the surface of the wheat leaves treated with KTP (**Figures 3**Aa, a’). The results showed that KTP significantly inhibited the formation of the mycelia structure of powdery mildew spores on the leaf surface.

Subsequently, we examined the samples at different infection times under an optical microscope. The germination growth of powdery mildew spores was a dynamic process (**Figures 3A**b–h). At 12 h, the powdery mildew spores of the *Bgt* group had already started to infect the leaves of wheat, and the spores germinated and produced a short primary germ tube, a process that involved the germination of the spores and the development of a germ tube. The germ tubes subsequently developed appressorium structures and underwent deformation to grow invading nails that penetrated the cell wall of the host epidermal cells, forming haustoria that parasitize the cytoplasm. Some of the spores began to grow secondary hyphae at 48 h to form mature mycelia, and at 144 h, the spores of the *Bgt* group all had notable hyphae (**Figure 3A**h). In contrast to those in the *Bgt* group (**Figures 3A**b’–h’), the spores in the KTP + *Bgt* group did not germinate long budding tubes at 12 h and did not form hyphal structures at 24 h; only a few spores those in at 144 h, and the hyphal growth state was significantly weaker than that of the *Bgt* group, while the remaining spores mostly remained in the appressorium status and did not grow exuberant hyphal structures. These results indicated that KTP delayed the germination of wheat powdery mildew spores.

In addition, the mature mycelium formation rate at 48 h was also determined in this test. As shown in **Figure 3B**, at 48 h after inoculation with powdery mildew, 19% of the germinated powdery mildew spores had germinated into mature mycelia in the KTP + *Bgt* group, which was significantly lower than that in the *Bgt* group (87%). In conclusion, KTP delayed the germination of powdery mildew spores from wheat and significantly inhibited the formation of mycelia of powdery mildew.

Besides, the morphologies of the wheat leaves infected with powdery mildew spores (144 h) were observed via SEM. **Figure 3C** shows that the mycelia of powdery mildew in the *Bgt* group grew vigorously and covered the surface of the wheat leaves, while the spores in the KTP + *Bgt* group were still in the state of an appressorium; moreover, an obvious mycelial structure was not observed. KTP was attached to the surface of the wheat leaves. The normally growing wheat leaves were covered with a layer of wax, while the wheat leaves of the KTP group had an uneven granular structure. Therefore, the above results indicated that the growth of powdery mildew spores was inhibited by the KTP nanoprotective membrane.

### 3.4 Differential gene expression analysis

PR1 is a defense protein involved in plant‒pathogen interactions. The expression of *PR1* is widely considered a reliable marker for activating the hypersensitivity (HR)-mediated defense pathway or establishing salicylic acid (SA)-mediated disease resistance in different plants (Xin et al., 2012). Chitinase plays an important role in plant defense against fungal pathogens and is also a type of pathogen-related protein (Ahmed et al., 2022). CHI1 protects plants from fungi by degrading fungal cell walls and inhibiting the growth of mycelia (Tayeh et al., 2015). Additionally, OXO is considered a marker of pathogen infection resulting from the interaction between powdery mildew and grains (Lane, 2002). OXO can produce H_2_O_2_, which is a kind of ROS (Christensen et al., 2004).

**Figure 4:**
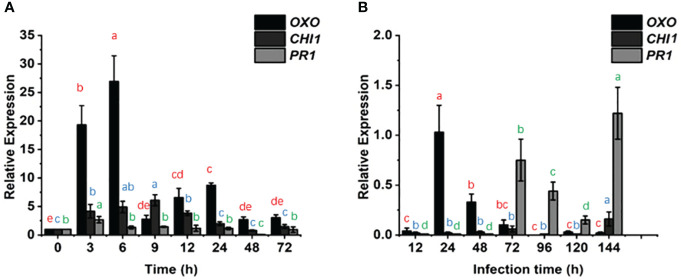
Effects of different experimental treatments on the expression of *OXO*, *CHI1* and *PR1* in wheat seedling leaves with time. **(A)** The transcription levels of *OXO*, *CHI1* and *PR1* in the KTP group and CK group were examined and compared by qRT-PCR. **(B)** The transcription levels of *OXO*, *CHI1* and *PR1* in the KTP + *Bgt* group and *Bgt* group were examined and compared by qRT-PCR. The expression of 3 genes in CK group and *Bgt* group were averaged as 1 (0).

We examined the changes in the *PR1*, *CHI1* and *OXO* genes over time after KTP spraying and powdery mildew invasion into wheat (**Figures 4A, B**). The relative transcript levels of *PR1* in the CK and KTP groups did not significantly differ at 0 h, 6-24 h, or 72 h. Except for at 144 h, the transcript level of *PR1* in the KTP + *Bgt* group was lower than that in the *Bgt* group throughout the measurement period. These findings indicated that KTP spray decreased *PR1* transcription in response to powdery mildew invasion and that KTP did not affect the transcript levels of *PR1* in normal wheat. Additionally, the expression of *CHI1* in the *Bgt* group was 16.67 times greater than that in the KTP + *Bgt* group (72 h). Taken together, these findings indicated that the transcript level of *CHI1* in wheat plants strongly promoted the production of chitinase to play a protective role against powdery mildew, while spraying KTP reduced the level of powdery mildew-related stress on the plants, so the transcript level of *CHI1* in the KTP + *Bgt* group was relatively low. However, there was no significant difference in the transcript levels of *CHI1* between the CK and KTP groups at 0 h, 24 h, 48 h and 72 h. These findings indicated that KTP did not affect the transcription of *CHI1* but rather reduced it by alleviating the infestation of wheat by powdery mildew. In addition, after spraying KTP or inoculating powdery mildew, the expression of *OXO* increased rapidly and then began to decline slowly. These findings suggested that the expression of *OXO* decreased with increasing time. Notably, at 72 h, the expression of *OXO* in the KTP group was 3 times that in the CK group, while that in the *Bgt* group was 10 times that in the KTP + *Bgt* group, and 50 times that at 144 h. Thus, KTP decreased the transcription of *OXO* induced by powdery mildew and played a protective role in wheat. The above data indicated that wheat infection with powdery mildew tended to increase the expression of *OXO* and subsequently induce the production of ROS to resist the invasion of powdery mildew. Therefore, these findings could help wheat to resist the invasion of powdery mildew, and reduce the expression of *PR* genes.

### 3.5 Biosafety Assessment of KTP

**Figure 5:**
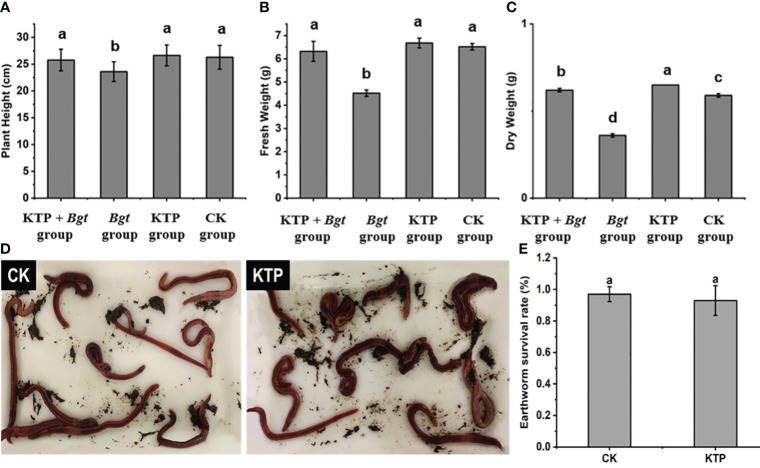
**(A)** Effects of different treatments on wheat plant height. **(B)** Effects of different treatments on wheat fresh weight. **(C)** Effects of different treatments on wheat dry weight. **(D)** Earthworm survival state. **(E)** Effect of KTP for earthworm survival.

To explore the effect of KTP on the growth of wheat, we measured the plant height, fresh weight and dry weight of each group of wheat plants for 7 days. The *Bgt* group yielded the shortest wheat plants, with a plant height of 23.60 cm, which was significantly lower than that of the other three groups (**Figure 5A**). The fresh weight and dry weight of the wheat plants in the *Bgt* group were 4.52 g and 0.36 g respectively, which were significantly lower than those in the other three groups (**Figures 5B, C**). Therefore, spraying KTP prevents a reduction in the fresh weight and dry weight of wheat plants caused by powdery mildew infection, and KTP has no side effects on the growth of wheat plants.

KTP might leach into the soil after rain, so the safety of KTP for soil organisms was evaluated. There was no significant difference in the survival status of earthworms in the KTP-treated soil compared to that in the CK group, in which survival rates were 93% and 97%, respectively (**Figures 5D, E**). It could be concluded that KTP had no effect on plants or soil organisms and had good biosafety.

## 4 Conclusion

Kaolin, TiO_2_-NPs, and liquid paraffin form KTP through the interaction of hydrogen bonding as well as electrostatic attraction. KTP delayed the germination of powdery mildew spores and inhibited the formation of mycelia. KTP has good physicochemical properties and meets field conditions, which could significantly reduce the occurrence of powdery mildew, thus preventing yield loss caused by powdery mildew. Therefore, this study provides an efficient, safe, and green management strategy for powdery mildew infection in large field areas.

## Data availability statement

The original contributions presented in the study are included in the article/supplementary material. Further inquiries can be directed to the corresponding author.

## Author contributions

HZ: Writing – review & editing, Conceptualization, Investigation, Methodology, Resources, Writing – original draft. MY: Writing – review & editing, Formal analysis, Investigation, Methodology, Writing – original draft. YG: Methodology, Writing – original draft, Formal analysis, Writing – review & editing. PS: Formal analysis, Writing – review & editing, Methodology, Resources, Writing – original draft. HJ: Writing – original draft, Formal analysis, Funding acquisition, Resources, Validation, Visualization, Writing – review & editing. CT: Writing – review & editing, Funding acquisition, Supervision, Validation, Visualization, Writing – original draft. HM: Investigation, Project administration, Writing – review & editing. LW: Writing – review & editing, Conceptualization, Resources, Supervision.
